# ATP-competitive mTOR kinase inhibitors delay plant growth by triggering early differentiation of meristematic cells but no developmental patterning change

**DOI:** 10.1093/jxb/ert242

**Published:** 2013-08-20

**Authors:** Marie-Hélène Montané, Benoît Menand

**Affiliations:** ^1^Aix-Marseille Université, Laboratoire de Génétique et Biophysique des Plantes, Marseille, F-13009, France; ^2^Centre National de la Recherche Scientifique, Unité Mixte de Recherche 7265 Biologie Végétale et Microbiologie Environnementales, Marseille, F-13009, France; ^3^Commissariat à l’Energie Atomique et aux Energies Alternatives, Institut de Biologie Environnementale et Biotechnologie, Marseille, F-13009, France

**Keywords:** *Arabidopsis*, ATP-competitive mTOR kinase inhibitors, cell size, differentiation, *Lotus*, meristem, millet, *Nicotiana*, rice, root growth, root hairs, target of rapamycin.

## Abstract

The TOR (target of rapamycin) protein, a large phosphatidylinositol 3-kinase-like protein kinase (PIKK) that is conserved in eukaryotes and is a central regulator of growth and metabolism. The analysis of function of TOR in plant growth and development has been limited by the fact that plants are very poorly sensitive to rapamycin. As the kinase domain of TOR is highly conserved, this study analysed the dose-dependent effect of three sets of first- and second-generation ATP-competitive inhibitors (called asTORis for active-site TOR inhibitors) recently developed for the human TOR kinase on *Arabidopsis thaliana* growth. All six asTORis inhibited plant root growth in a dose-dependent manner, with 50% growth inhibitory doses (GI_50_) of <10 μM and <1 μM for the first- and second-generation inhibitors, respectively, similarly to the values in mammalian cells. A genetic approach further demonstrated that only asTORis inhibited root growth in an *AtTOR* gene-dosage-dependent manner. AsTORis decreased the length of: (i) the meristematic zone (MZ); (ii) the division zone in the MZ; (iii) epidermal cells in the elongation zone; and (iv) root hair cells. Whereas meristematic cells committed to early differentiation, the pattern of cell differentiation was not affected *per se*. AsTORis-induced root hair growth phenotype was shown to be specific by using other growth inhibitors blocking the cell cycle or translation. AsTORis dose-dependent inhibition of growth and root hairs was also observed in diverse groups of flowering plants, indicating that asTORis can be used to study the TOR pathway in other angiosperms, including crop plants.

## Introduction

How plant and plant-cell growth is regulated, and particularly how regulatory systems inherited from the eukaryotic and prokaryotic ancestors of plants are integrated, are still open questions (Robaglia *et al.*, 2012). Target of rapamycin (TOR) is a protein kinase that controls growth and metabolism in response to environmental cues in eukaryotes (Loewith and Hall, 2011; Laplante and Sabatini, 2012). TOR belongs to the family of phosphatidylinositol 3-kinase-like protein kinases (PIKKs) that includes the checkpoint kinases ATM (ataxia telangiectasia mutated) and ATR (ATM- and RAD3-related protein) (Templeton and Moorhead, 2005). The TOR pathway is the focus of large research efforts in yeast and animals due to its role in health, disease, cancer, and aging.

In both yeast and mammals, TOR, which is a very large protein, is present in at least two different multi-protein complexes, TORC1 and TORC2, that regulate different outputs required for cell and organism growth (Laplante and Sabatini, 2012). When active, yeast and mammalian TORC1 positively regulate protein synthesis, cell-cycle progression, and energy metabolism, while inhibiting stress responses, such as autophagy. The best-characterized targets of mammalian TORC1 are S6 kinase 1 (S6K1), which phosphorylates the ribosomal protein S6, and eukaryotic initiation factor 4E-binding protein 1 (4E-BP1), which regulates the initiation of cap-dependent translation (Laplante and Sabatini, 2012). The main known function of TORC2 in yeast and mammals is to regulate spatial control of cell growth via the actin cytoskeleton. TORC1 and TORC2 regulate these important cellular processes in response to the environmental conditions perceived by the cell. In yeast, TORC1 is activated by nutrients through interactions with the vacuole, while starvation for carbon, nitrogen, phosphate, or amino acids mimics TORC1 inhibition (Loewith and Hall, 2011). Various stresses, including high salt, oxidative stress, and high temperature, are also sensed by TORC1 and TORC2. In addition, systemic signalling in multicellular organisms is integrated by the mTOR pathway (Laplante and Sabatini, 2012). Indeed, TORC1 and TORC2 are activated by insulin-like growth factors through the phosphatidylinositol 3- kinase (PI3K) pathway. Furthermore, TORC1 is inhibited by adenosine monophosphate-activated protein kinase (AMPK), the activity of which depends on the cell energy status (Laplante and Sabatini, 2012).

Complete genome sequence analysis indicates that some but not all of the proteins involved in the mammalian TOR pathway are found in all six major eukaryotic groups (Serfontein *et al.*, 2010). These conserved proteins, PI3K, Phosphatase and TENsin homologue (PTEN), TOR, regulatory-associated protein of mTOR (RAPTOR), AMPK and S6K, may form the ancient eukaryotic TOR signalling pathway that has integrated particular inputs and outputs during the divergent evolution of each group. The finding that *AtTOR* loss-of-function mutants are embryo lethal and that *AtTOR* is expressed in meristems first indicated that the TOR pathway is essential for plant growth (Menand *et al.*, 2002). Transgenic lines carrying induced RNA silencing of *AtTOR* experience impaired post-embryonic growth, a decrease in the ratio of polysomes to monosomes, lipid changes, and altered sensing of abiotic stresses (Deprost *et al.*, 2007; Caldana *et al.*, 2012; Xiong and Sheen, 2012). Even though these data validate the important role of AtTOR in growth regulation, such inducible *AtTOR* RNA-silencing lines are not easy to handle as they do not permit quantitative and kinetically controlled modulation of growth and/or AtTOR levels.

In yeasts and animals, rapamycin, which is produced by *Streptomyces hygroscopicus*, has been used extensively to dissect the TOR pathway due to its ability to specifically inhibit TOR activity through the formation of a ternary complex with TOR and the peptidyl-prolyl *cis*-*trans* isomerase FKBP12 (FK506 and rapamycin-binding protein of 12kDa) (Loewith and Hall, 2011). The use of rapamycin in plants is limited as different authors have reported that rapamycin does not affect *Arabidopsis thaliana* wild-type (WT) organ growth, even at concentrations up to the tens of micromolar range in solid medium (Sormani *et al.*, 2007; Leiber *et al.*, 2010; Ren *et al.*, 2012). As *A. thaliana* FKBPs do not carry the amino acids critical for the interaction with rapamycin in animals and yeast, different groups have overexpressed yeast or mammalian FKBP12 proteins to create plants sensitive to rapamycin (Sormani *et al.*, 2007; Leiber *et al.*, 2010; Ren *et al.*, 2012; Xiong and Sheen, 2012). These transgenic lines showed a partial inhibition of organ growth in response to rapamycin, but, in the sole study where plants were treated with a range of rapamycin concentrations, no clear-cut dose-dependent effect of rapamycin on growth was reported (Ren *et al.*, 2012). Indeed, a plateau value of growth inhibition of about 50% was observed along the concentration range from 0.5 to 22 μM, which was associated with a phenotype of shortened root hairs (Ren *et al.*, 2012). A similar phenotype was also reported for *A. thaliana* seedlings germinated in liquid medium with 10 μM rapamycin (Xiong and Sheen, 2012). However, these phenotypes observed under different growth conditions are hard to compare, preventing easy conclusions.

In addition, rapamycin only partially inhibits TORC1 and does not inhibit TORC2 in mammals (Feldman and Shokat, 2011; Laplante and Sabatini, 2012). Furthermore, unexpected molecular phenotypes unrelated to the AtTOR pathway might be generated by heterologous expression of FKBP12s due to its peptidyl-prolyl *cis*-*trans* isomerase activity (Gerard *et al.*, 2011). Progress on studies of the TOR pathway depends strongly on the availability of specific and workable tools to manipulate its activity.

New mammalian TOR-specific inhibitors have been developed recently by several groups with the purpose of inhibiting the TOR pathway more efficiently than rapamycin for cancer therapy (Zhang *et al.*, 2011). All these new TOR inhibitors are ATP competitive, as they target the ATP-binding pocket of the kinase domain and are called active-site TOR inhibitors (asTORis) (Dowling *et al.*, 2010). They have been selected for their specificity by *in vitro* kinase assays with a wide range of protein kinases (Garcia- Martinez *et al.*, 2009; Thoreen *et al.*, 2009; Chresta *et al.*, 2010; Garcia- Martinez *et al.*, 2009; Q. Liu *et al.*, 2010; Yu *et al.*, 2009, 2010; Liu *et al.*, 2011). In this study, we showed that asTORis clearly inhibit whole-plant growth in a concentration- and *AtTOR* gene-dosage-dependent manner. The phenotype of root inhibition is reported, i.e. reduction in organ growth, as well as early differentiation of meristematic cells leading to meristem size reduction and shortening of epidermal cells and root hairs without changes in the pattern of differentiation. We also showed that asTORis are potent and robust inhibitors in diverse angiosperms, including crops.

## Material and methods

### Plant material

*A. thaliana* WT plants used were from Columbia (Col-0) or Wassilewskija (WS) ecotypes. The ecotype used was Col-0, unless specified otherwise. The *TOR*/*tor-1* (WS)*, TOR*/*tor-3* (Col-0), *rhd6-2* (Lansberg erecta) and *CYCB1;1:*GUS (Col0) lines have been described previously (Colon-Carmona *et al.*, 1999; Menand *et al.*, 2002, 2007; Ren *et al.*, 2011). *Lotus japonicus* cv. Gifu seeds were a gift from C. Vriet and T.L. Wang (John Innes Centre, Norwich, UK). *Nicotiana benthamiana* seeds were from the Tobacco Institute, SEITA, Bergerac, France). *Panicum miliaceum* (‘millet brun’) seeds were purchased from Moulin Meckert-Diemer (Krautwiller, France). *Oryza sativa* (cv. Nipponbare) seeds were from S. Jouannic (IRD, Montpellier, France).

### In vitro plant growth

All products were purchased from Sigma unless stated otherwise. Seeds of all species were germinated and grown on a solid medium containing 5mM KNO_3_, 2.5mM KH_2_PO_4_, 2mM Mg(SO_4_)_2_, and 2mM Ca(NO_3_)_2_ as described by Estelle and Somerville(1987) with the microelements of Santoni *et al.* (1994) designed for *A. thaliana*. The medium contained 1% glucose with the pH adjusted to 5.5 and was solidified by 4g l^–1^ of Phytagel^TM^ (see details in Supplementary Fig. S2 at *JXB* online) before autoclaving at 115 °C for 20min. The ammonium iron (III) citrate was added after autoclaving from a 2% stock solution that had been filter sterilized (0.22 μm). Plates were carefully poured and protected from desiccation under the flow bench. Transfer plates containing filter-sterilized DMSO at a final concentration of 0.1% with or without drug were stored in the dark for up to 1 week in plastic bags.

In all cases, 0.7% Tween 20 was added to the seed sterilization solution. *A. thaliana* and *N. benthamiana* seeds were surface sterilized for 10min in a solution containing 90% ethanol, 0.8 % sodium dichloroisocyanurate dihydrate (SDCD; 02 Javel-pastille, Richet, France) and then washed twice in absolute ethanol. *L. japonicus* seeds were surface sterilized in 0.8 % SDCD for 10min and washed twice in water. *P. miliaceum* seeds were surface sterilized in water containing 0.1% calcium hypochloride for 15min and washed six times with autoclaved water. *O. sativa* seeds were surface sterilized in a solution of 20% sodium hypochlorite and washed six times with water. After sowing on plates, *A. thaliana* seeds were incubated for 2 d at 4 °C in the dark before germination. After surface sterilization, *O. sativa* seeds were incubated in sterile water overnight at room temperature in the dark before plating. *L. japonicus*, *N. benthamiana*, and *P. miliaceum* seeds were sown and transferred to the growth chamber directly after surface sterilization. Seeds were germinated on solid medium for 2 d (*P. miliaceum* and *O. sativa*), 3 d (*A. thaliana*), 4 d (*L. japonicus*), or 5 d (*N. benthamiana*) before transfer to drug-containing medium, i.e. when the root length was <1cm. For *O. sativa* and *P. miliaceum*, plantlets were transferred to square Petri dishes (120×120×17mm) containing 50ml of solidified medium, whereas 90mm diameter round Petri dishes containing 25ml of medium were used for the other species. The Petri dishes were placed vertically to allow the roots to grow on the surface of the medium. Photoperiod of 16h at 23 °C (80 μmol photons m^–2^ s^–1)^ was followed by 8h of dark at 18 °C.

### Drug treatment

The suppliers and characteristics of the drugs are listed in Table 1. All drugs were stored and dissolved in DMSO according to the manufacturer’s instructions. Aliquots of stock solutions were stored at –20 °C at a concentration of 10 to 60mM depending on the asTORi. Plates containing drugs were generally prepared a couple of days before use. Plants were carefully transferred with jeweller’s forceps onto new plates containing drugs or DMSO. Details are given in Supplementary Fig. S2.

**Table 1.** Characteristics of the drugs used in this study

Drug name	Source	Target	Type of inhibition	GI_50_ in animal cells (μM)^a^	Selectivity	Tested in plants	Reference(s)	No. kinases tested
WYE-354	Chemdea	mTOR	ATP competitive	0.3–1		No	Yu *et al.* (2009)	260
WYE-132	Chemdea	mTOR	ATP competitive	0.01		No	Yu *et al.* (2010)	
KU-0063794	Tocris Biosciences	mTOR	ATP competitive	2.5–10	At least 1000-fold specificity over other PIKKs and PI3-Ks	No	Garcia-Martinez *et al.* (2009); Chresta *et al.* (2010); Syed *et al.* (2013)	76
AZD-8055	Chemdea	mTOR	ATP competitive	0.03–0.1	At least 1000-fold specificity over other PIKKs and PI3-Ks	No	Chresta *et al.* (2010)	70
Torin1	Gift of Dr N. Gray and Tocris Biosciences	mTOR	ATP competitive	<0.25	>300-fold selectivity over PI3K and PIKK	No^b^	Thoreen *et al.* (2009); Q. Liu *et al.* (2010)	442
Torin2	Gift of Dr N. Gray and Tocris Biosciences	mTOR	ATP competitive	0.01–0.2	800-fold selectivity over PI3K	No	Liu *et al.* (2011; 2012b)	440
QL-IX-55	Gift of Dr N. Gray	*S. cerevisiae* TORC1	ATP competitive	0.16		No	Liu *et al.* (2012*b*)	440
KU-55933	Tocris Biosciences	ATM	ATP competitive	10	No TORC1 and C2 inhibition	No^c^	Golding *et al.* (2009); Li and Yang (2010)	229
KU-60019	Tocris Biosciences	ATM	ATP competitive	0.1–0.3	High below 1μM	No	Golding *et al.* (2009)	229
PI-103	Tocris Biosciences	PI 3-K /mTORC1	ATP competitive	0.1–0.5	High below 10 μM	No	Fan *et al.* (2006); Raynaud *et al.* (2007)	
LY294002	Tocris Biosciences	Broad-spectrum PI3-K inhibitors; mTORC1	ATP competitive	4–15; 20–40	Serum dependent	Yes	Gharbi *et al.* (2007); Lianguzova *et al.* (2007); Raynaud *et al.* (2007); Moon *et al.* (2009); Finlay and Griffin (2012)	
Cycloheximide	Sigma	Protein synthesis	Fixation on the E-site of ribosome	1	ND	Yes	X. Liu *et al.* (2010; Schneider-Poetsch *et al.* (2010)	
Roscovitin	Sigma	Pan CDK and pyridoxal kinase	ATP competitive	10–50	Tenfold higher than for ERK8	Yes	Meijer *et al.* (1997); Planchais *et al.* (1997); Bach *et al.* (2005); Bain *et al.* (2007)	
Rapamycin	LC labs	TOR (only TORC1)	Allosteric through the formation of a complex with TOR and FKBP12	0.005–20		Yes	Sormani *et al.* (2007); Ren *et al.* (2012); Xiong and Sheen (2012) Syed *et al.* (2013)	

ND, not determined.

^*a*^Concentration strongly depends on mammalian cell type.

^*b*^In plants, the drug was only used in biochemical measurements and growth inhibition was not reported (Schepetilnikov *et al.*, 2011; Xiong *et al.*, 2013).

^*c*^The drug was only used to increase γH2AX foci in plant nuclei (Amiard *et al.*, 2010).

### Root growth measurements

The position of the tip of each root was labelled every day on the back of the Petri dish. Six days after transfer to the new medium, all Petri dishes were scanned (EPSON Perfection 1250, EPSON TWAIN5 software) and root growth was measured with ImageJ software. Dose–response curves were expressed relative to the control growing on DMSO. The time course of the effect of inhibitors was expressed as the ratio of each daily length of treated roots compared with the corresponding length of the control growing on DMSO.

### Microscopy

Pictures of the aerial parts of plants and the differentiated zone containing root hairs were taken directly from the Petri dish with a Zeiss stereomicroscope (Stereo Discovery V12). For meristem observations, roots were mounted in chloral hydrate solution (100g of chloral hydrate, 30ml of water, and 5ml of glycerine) and observed with a Zeiss microscope Axo imager M2 with a differential interference contrast illumination system. Cell surface area, cell length, and root hair measurements were done with ImageJ software and expressed relative to the control growing on DMSO, unless otherwise specified. The size of the meristematic zone (MZ), corresponding to the distance between the quiescent centre and the first elongated cortical cell, was measured as described previously (Perilli and Sabatini, 2010).

### β-Glucuronidase (GUS) staining

Plants were incubated at 37 °C in GUS staining solution (1mM 5-bromo-4-chloro-3-indolyl-glucuronide, 0.5mM potassium ferricyanide, 0.5mM potassium ferrocyanide, and 10mM sodium phosphate buffer, pH 7) for 2h for *CYCB1;1:*GUS, 4h for *rhd6-2*, and 16h for *TOR*/*tor-1* lines.

### Characterization of the progeny of TOR/tor-1 and TOR/tor-3 heterozygotes

The *tor-1* and *tor-3* mutants are embryo lethal, so the progeny of heterozygous plants produce only WT and heterozygous plants (Menand *et al.*, 2002; Ren *et al.*, 2011). As the *tor-1* allele carries a translational fusion between the TOR N-terminal sequence and GUS, leading to GUS expression in meristems (Menand *et al.*, 2002), we used GUS staining to distinguish *TOR/tor-1* (GUS positive) and *TOR*/*TOR* (GUS negative) plants at the end of the growth measurements. The progeny of *TOR*/*tor-3* plants were genotyped by PCR using combinations of the primers Tor-3R (5′-GGCAGTCAAACTATCAGCCTG-3′)/Tor-3L (5′-TGTCCCT GTAGATTGCTCCAC-3′) and Tor-3R/LBa1 (5′-TGGTTCACGT AGTGGGCCATCG-3′), as described previously (Ren *et al.*, 2011).

## Results

### asTORis inhibit plant root growth in A. thaliana

**Fig. 1.**
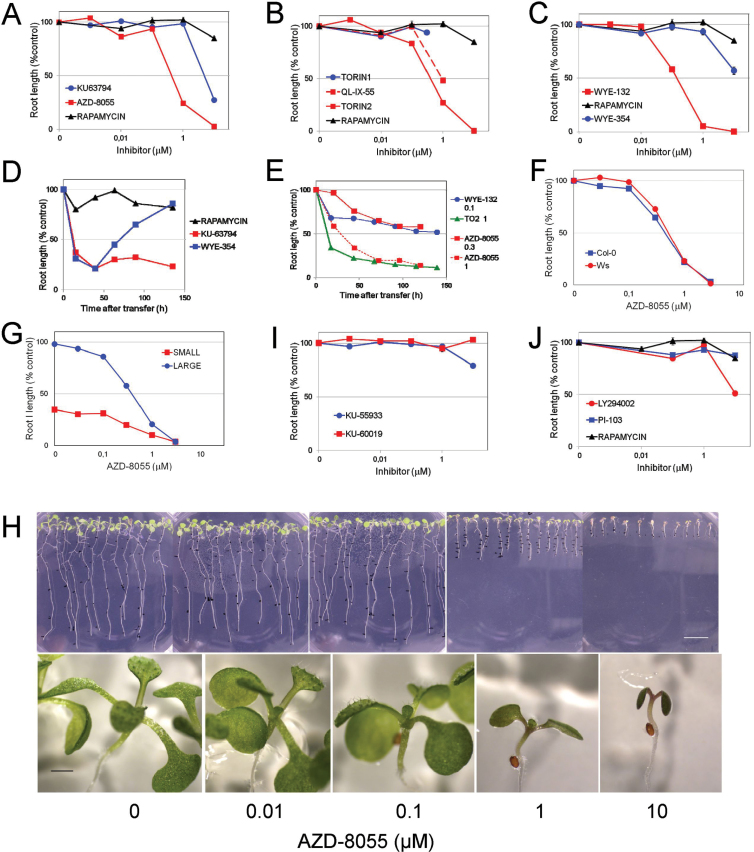
Inhibition of *A. thaliana* root growth by ATP-competitive inhibitors of TOR, ATM, and PI3Ks. Plants were grown for 3 d without drug or DMSO, transferred to drug-containing plates and grown for 6 d. (A–C) Dose-dependent effect of three sets of first-generation (blue) and second-generation (red) asTORis on primary root growth. Rapamycin (black) was tested at the same final concentration of 0.1% DMSO. (D–E) Time course of root growth delay of two first-generation asTORis (10 μM), compared with 10 μM rapamycin (D) and second-generation asTORis at the μM concentration indicated (E). (F, G) Dose–response curves of AZD-8055 on ecotypes WS and Col-0 (F) and on Col-0 plants germinated on medium with lower glucose (0.15% instead of 0.8%) to get a bimodal plant population size distribution (G). (H) Pictures of whole plants (upper panel) and aerial parts (lower panel) after growth on different AZD-8055 concentrations. Bars, 1mm (lower panel), 1cm (upper panel). (I) Dose-dependent effect of first-generation (blue) and second-generation (red) asATMis on root growth. (J) Dose-dependent effect of the PI3Ks inhibitors PI-103 (blue) and LY294002 (red) on root growth compared with rapamycin (black). Data are the mean of two experiments done with two or three plates containing ten seeds. The standard error was low (~2%) and is often hidden by the size of points.

The amino acid residues of the TOR kinase domain that are important for ATP binding are largely conserved among *A. thaliana* and mammals (Supplementary Fig. S1 at *JXB* online). As new ATP-competitive inhibitors highly specific of mTOR have been developed (Zhang *et al.*, 2011), we tested the dose-dependent effect of three sets of these asTORis on *A. thaliana* root growth. The main characteristics of the drugs used in our study are summarized in Table 1. Each set contained a first-generation inhibitor, namely KU63794, Torin1, and WYE-354 (Garcia-Martinez *et al.*, 2009; Thoreen *et al.*, 2009; Yu *et al.*, 2009) and a second-generation counterpart that was developed to improve pharmacodynamics, namely AZD-8055, Torin2 and WYE-132, respectively (Chresta *et al.*, 2010; Yu *et al.*, 2010; Liu *et al.*, 2011). We developed a sensitive and robust growth inhibition assay to test the effect of DMSO-dissolved drugs on primary root growth, as described in Supplementary Fig. S2. The dose–response curves for the effects of these drugs on primary root length showed that each asTORis inhibited root growth at concentrations ranging from 0.1 up to 10 μM. In contrast, rapamycin had almost no inhibitory effect on root growth over this concentration range (Fig. 1A–C). The second-generation molecules were about ten times more efficient at inhibiting growth than the first-generation inhibitors, in agreement with the results reported for mammalian cells (Table 1). Indeed, all second-generation drugs designed for mammals or for yeast (QL-IX-55; Liu *et al.*, 2012*b*) (Fig. 1B) clearly inhibited root growth at concentrations below 1 μM. Moreover, the time course of growth delay showed that all drugs except for WYE-354 triggered a rapid, constant, and reproducible level of growth reduction in a concentration-dependent manner as exemplified in Fig. 1D and E. The higher the concentration, the sooner the plateau value of inhibition was reached. These data indicated that the bioavailability and pharmacokinetics of the drugs tested enable their use in plant growth inhibition studies. This was not the case for rapamycin and WYE-354. These inhibitors easily precipitated in the medium at concentrations above 10 μM during the growing period and their growth inhibition capacity was lost after 1–2 d (Fig. 1D).

AZD-8055, one of the potent second-generation asTORis, inhibited the growth of two ecotypes of *A. thaliana* (Fig. 1F) and of plant populations whose average size was ~1cm (large) or 0.5cm (small) before transfer to drug-containing medium (Fig. 1G). As the same 50% growth inhibitory dose (GI_50_) was observed, this suggested that asTORis impair growth independently of the plant size and of the ecotype. Concomitantly with roots shortening, the cotyledons and leaves stayed green and their development was severely delayed by AZD-8055 (Fig. 1H). These data suggested that the delayed growth at the whole-plant level might be due to a decreased meristem activity and possibly the reduced size of differentiated cells. In addition, growth was rescued when plants were transferred back to medium without drug, showing the reversibility of the effect of the drug.

AZD-8055 is at least a 1000-fold less potent towards the related PI3K and the PIKK kinases ATM and DNA-PK than TOR in mammalian cells (Chresta *et al.*, 2010). Recently, ATP-competitive inhibitors of ATM (asATMis), another PIKK conserved between *A. thaliana* and *Homo sapiens* (Garcia *et al.*, 2000), were developed for mammalian cells (Golding *et al.*, 2009). The first- and second-generation asATMis KU-55933 and KU-60019, respectively, have similar if not identical ATM target specificity with little to no non-specific target effects and, notably, they do not inhibit mTOR (Golding *et al.*, 2009). KU-55933 was reported to be active in plants through inhibiting γ-H2AX protein phosphorylation (Amiard *et al.*, 2010) and *AtATM* mutants have no growth defect. We wished to check whether asATMis could affect plant growth. No growth inhibition was observed (Fig. 1I), indicating that asATMis action does not interfere with the asTORis action on growth inhibition. This suggested that the selectivity of the active-site inhibitors of ATM and mTOR is probably conserved towards the corresponding kinases in *A. thaliana* for a large range of concentrations.

We also tested the dose-dependent effect on growth of two ATP-competitive inhibitors of mammalian PI3K, LY294002 and PI-103 (Table 1). LY294002, which was reported to be active also in plants (Lee *et al.*, 2008), is a broad-spectrum inhibitor of PI3Ks that was not found to be exclusively selective for PI3K as it also inhibits other kinases including mTOR (Gharbi *et al.*, 2007). PI-103 inhibits PI3K and mTORC1 (Fan *et al.*, 2006; Raynaud *et al.*, 2007). The effect of PI-103 could not be established as it slightly precipitated in the medium. LY294002 inhibited growth with a GI_50_ dose in the range of 10 μM (Fig. 1J), very close to that inhibiting growth of mammalian cells (Jiang *et al.*, 2010).

Altogether, the ATP-competitive inhibitors of PI3K and PIKKs (ATM and TOR) designed for mammalian kinases that were workable in our conditions triggered plant growth inhibition (LY294002 and asTORis) or not (asATMis), with a specificity similar to that in mammalian cells. These results strongly suggest that the efficiency and specificity of kinase inhibition by ATP-competitive inhibitors is similar between plants and mammals.

### AsTORis act in a *TOR* gene-dosage-dependent and specific manner in A. thaliana

**Fig. 2.**
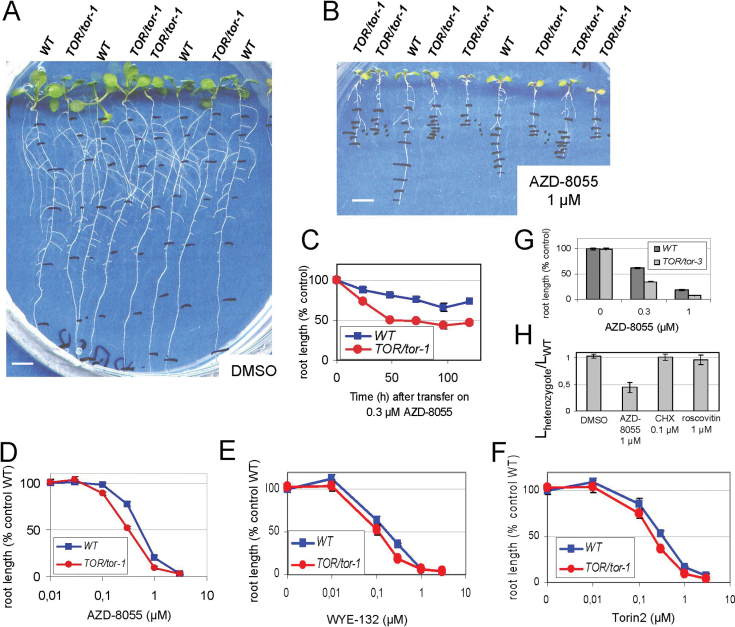
AZD-8055, WYE-132, and Torin2 induce *AtTOR* haplo-insufficiency. Pictures and measurements were done 6 d after transfer of 3-d-old plants onto medium containing the indicated concentration of AZD-8055. (A, B) Whole plants from the progeny of *TOR*/*tor1* heterozygotes grown vertically on DMSO (A) or 1 μM AZD-8055 (B). The genotypes of plants are indicated at the top of each plant after GUS expression was determined for each plant, whose growth was previously recorded. Bars, 500 μm. (C) Time course of root growth delay by 0.3 μM AZD-8055 of *TOR*/*tor-1* and WT. (D–F) Dose-dependent effect of AZD-8055 (D), WYE-132 (E), and Torin2 (F) on the growth of *TOR*/*tor1* and WT plants. (G) Differential sensitivity to AZD-8055 of root growth of *TOR*/*tor-3* plants compared with WT. (H) Ratio of the root lengths of the *TOR*/*tor1* on WT plants grown on DMSO or on the growth inhibitors AZD-8055, cycloheximide (CHX), or roscovitine at the indicated concentrations.

Studies in yeast and mammalian cells have demonstrated that lowering the dosage of a single gene encoding the target of a drug from two copies in a homozygous strain to one copy in a heterozygous strain results in increased drug sensitivity. This phenomenon of induced haplo-insufficiency has been used to identify the biological targets of drugs (Giaever *et al.*, 1999; Lum *et al.*, 2004; Kung and Shokat, 2005). To determine whether asTORis induce *AtTOR* haplo-insufficiency, we tested the dose-dependent effect of AZD-8055, WYE-132 and Torin2 on the growth of heterozygous *AtTOR* mutants. Loss-of-function homozygous mutants in the *AtTOR* gene are embryo lethal, but no phenotype has been described previously in heterozygous plants (Menand *et al.*, 2002; Ren *et al.*, 2011). The *GUS* reporter gene was fused in frame downstream of the coding sequence of the *AtTOR* gene, so the the *tor-1* allele could be detected by following *GUS* expression in meristems (Menand *et al.*, 2004). The progeny of *TOR*/*tor-1* heterozygous plants that had been recorded individually for daily growth for 6 d were each subjected to GUS staining for genotyping. *TOR*/*tor-1* heterozygous plants were not significantly different from WT when grown without the drug (Fig. 2A), whereas they were significantly smaller than WT plants when transferred to medium containing asTORis, as shown for AZD-8055 in Fig. 2B. Indeed, while the progression of the time course of inhibition was the same for WT and heterozygotes (Fig. 2C), the GI_50_ value was twofold lower in *TOR*/*tor-1* plants than in WT (Fig. 2D–F). This suggested that all three second-generation asTORis could be used interchangeably. We performed a similar experiment using *TOR*/*tor-3*, another independent line that has a T-DNA insertion at the beginning of the *TOR* coding sequence (Ren *et al.*, 2011). In a similar manner to *TOR*/*tor1*, *TOR*/*tor-3* heterozygous plants, which were genotyped by PCR, grew like WT plants without drug and showed increased sensitivity compared with the WT when treated with AZD-8055 (Fig. 2G). Taken together, these data showed that the level of root growth inhibition by second-generation asTORis is dependent on the number of copies of the *AtTOR* gene.

To show that the heterozygote hypersensitivity phenotype was exclusively dependent on asTORis, we tested the dose-dependent effect of other growth inhibitors that act on different intracellular targets. Cycloheximide, an inhibitor of protein synthesis, and roscovitine, a cyclin-dependent kinase inhibitor, were selected because they were previously tested in plants and mammalian cells (Wallace *et al.*, 1970; Planchais *et al.*, 1997). They both inhibited root growth in a concentration-dependent manner (Supplementary Fig. S3 at *JXB* online) and were stable over time (Supplementary Fig. S4 at *JXB* online). We observed no difference in sensitivity to cycloheximide and roscovitine between *TOR*/*tor-1* heterozygous and WT plants (Fig. 2H). Therefore, these two compounds did not induce the haplo-insufficiency phenotype, revealing the selectivity of asTORis towards AtTOR.

Altogether, these data strongly suggest that asTORis-induced haplo-insufficiency is exclusively linked to the *AtTOR* gene and that asTORis inhibit root growth via inhibition of the AtTOR protein. As the *AtTOR* gene is unique and the GI_50_ dose of asTORis was twice as low for heterozygotes, it is tempting to suggest that the amount of TOR protein is twofold lower in heterozygotes. Therefore, whereas *TOR*/*tor* heterozygotes grow similarly to WT without asTORis, we assume that the reduced amount of TOR protein per se is not critical for growth.

### AZD-8055 induces exit from the MZ and inhibits cell growth and root hair elongation

The *A. thaliana* primary root is spatially separated into three developmental zones; the MZ is where new cells are produced through active proliferation, and they increase in size progressively until they reach the elongation zone (EZ), where the cells elongate rapidly, and the differentiation zone (DZ), where root hairs develop by tip growth from epidermal cells (Dolan and Davies, 2004).

**Fig. 3.**
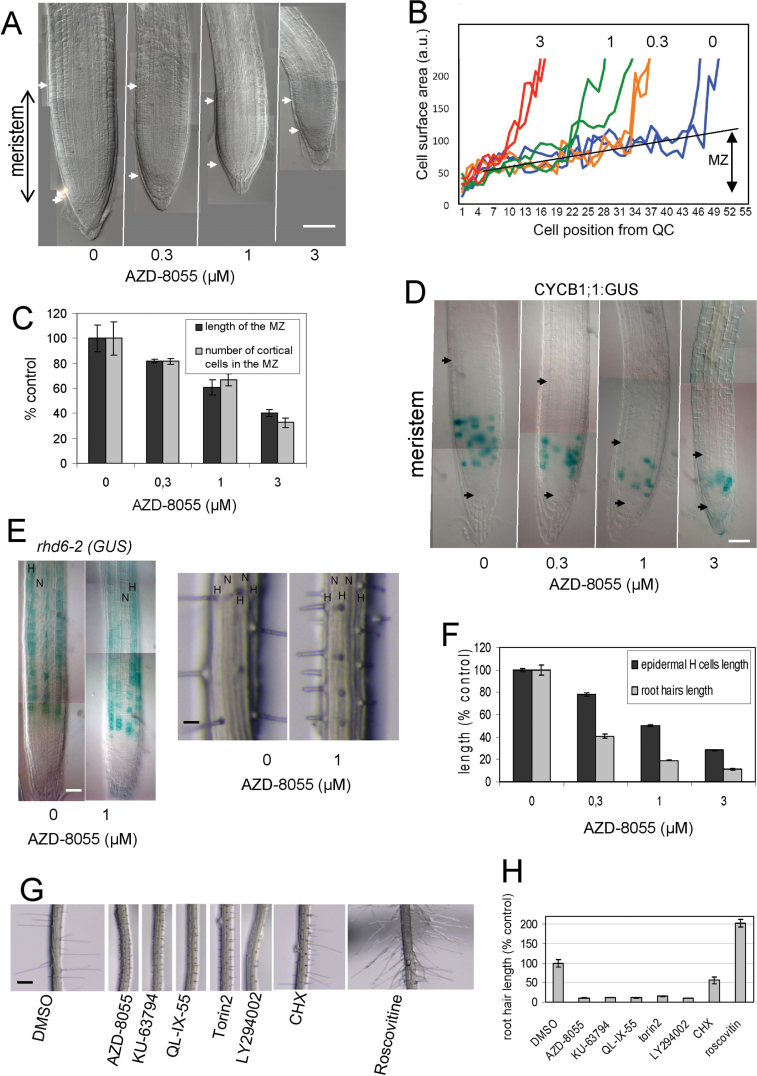
AsTORis induce *A. thaliana* root phenotypes. Pictures and measurements were done 2 d after transfer of 3-d-old plants onto medium containing the indicated concentrations of AZD-8055. (A) Differential interference contrast microscopy picture of the MZ delimited by the arrows. (B) Cell surface area distribution of cortical cells relative to the quiescent centre (QC). Individual cells of two roots were measured for each indicated concentration of AZD-8055 (μM). (C) Length of the MZ and number of cortical cells of the MZ. (D) Expression of the marker *CYCB1;1:GUS*. (E) Expression of the *rhd6-2* enhancer trap *GUS* gene (left panel) and pictures of epidermal cells in the differentiated area containing mature root hairs (right panel). Non-hair (N) and hair (H) cell files are indicated. (F) Dose response for AZD-8055 on the length of epidermal H cells and of root hairs. (G, H) Root hair phenotypes of plants grown with GI_50_ doses of the four indicated asTORis and LY294002 compared with cycloheximide (CHX) and roscovitine (G) and corresponding measurement of the inhibition relative to the DMSO control (H). Bars, 50 μm (A, D, E); 200 μm (G).

Two days after transfer of 3-d-old plants to AZD-8055, microscopic observations of root tips showed that AZD-8055 reduced the size of the MZ in a dose-dependent manner (Fig. 3A). Indeed, analysis of the surface area of cortical cells along the root, which reflects cell size, showed that the higher the AZD-8055 concentration, the smaller the size of cells leaving the MZ (Fig. 3B). In addition, the length of the MZ was linearly correlated with the number of cortical cells within the MZ (Fig. 3C). Therefore, the diminution of the MZ was due to a reduction in both the number of dividing cells and in cell growth in the MZ. CYCB1;1 is a marker of the G2/mitotic phase of the cell cycle and is expressed within the MZ in the zone of high mitotic activity (Colon-Carmona *et al.*, 1999). CYCB1;1 accumulates when the cell cycle is blocked after stress revealing an increase in the number of cells whose division is blocked by G2 checkpoints (Ricaud *et al.*, 2007). Such cell-cycle changes can be identified in plants expressing *CYCB1;1:GUS* by observing the density of blue cells. In *CYCB1;1:GUS* plants, we observed that increasing AZD-8055 reduced the zone of high mitotic activity (Fig. 3D). As the density of blue cells hardly changed within the zone of high mitotic activity, this suggested that G2 checkpoints that normally occur in a proliferating tissue are not impaired by asTORis. Altogether, our study indicated that AZD-8055 inhibits the cell proliferation capacity of the meristem by decreasing the number of cells in the MZ, mainly through promoting the acropetal differentiation of meristem cells.

In *A. thaliana* roots, epidermal cells are arranged in alternative files of hair-bearing (H) and non-hair-bearing (N) cells. *ROOT HAIR DEFECTIVE6* (*RHD6*) is a marker of H cells in the EZ before they produce hairs (Menand *et al.*, 2007). The pattern of *RHD6* expression in the EZ, and the alternation of H and N cell files in the differentiated root were also not affected by AZD-8055 (Fig. 3E). Cell size measurements in the DZ showed that AZD-8055 inhibited the length of H epidermal cells and of root hairs in a dose-dependent manner with a stronger effect on root hairs (Fig. 3 F). This indicated that AZD-8055 inhibits both the elongation of epidermal cells in the EZ and the root hair elongation that occurs in the DZ. Notably, the position of root hair emergence along epidermal cells was not affected (Supplementary Fig. S5 at *JXB* online). In summary, AZD-8055 induces MZ cells to differentiate and inhibits cell elongation and root hair growth but does not appear to affect the pattern of development per se.

Plants treated with four asTORis at concentrations close to their root GI_50_ showed a 90% shortening of root hairs (Fig. 3G, H). The effect of LY294002 was similar to that of asTORis whereas cycloheximide treatment resulted in a slight shortening of root hairs with an irregular distribution along the root. Conversely, roscovitine treatment resulted in very long root hairs. Therefore, we showed that several asTORis induced the same short root hair phenotype. This phenotype was distinct and separate from the root hair phenotypes caused by drugs that inhibit growth through blocking translation (cycloheximide) or the cell cycle (roscovitine). This evidence supports the notion that asTORis designed for mTOR specifically target AtTOR in *A. thaliana*. As LY294002, a broad-spectrum inhibitor of the kinase mPI3K that acts upstream of mTOR, induced the same root phenotype as asTORis, this suggests that the link between PI3K and TOR might be conserved in animals and plants.

### AsTORis inhibit growth of diverse angiosperms

Angiosperms evolved approximately 150 million years ago and rapidly expanded to form the largest group of plants (Soltis *et al.*, 2005). Angiosperms include monocots and eudicots. We selected species representative of diverse groups of angiosperms to test whether the sensitivity to asTORis was conserved. We selected two monocots, *O. sativa* (rice) and *P. miliaceum* (millet), and representatives of the two major clades of eudicots: the Rosids and Asterids. In addition to the Brassicale *A. thaliana*, *L. japonicus*, a second representative member of the Rosids, was chosen among the Fabales. We used the Solanales *N. benthamiana* as a representative of the Asterids.

**Fig. 4.**
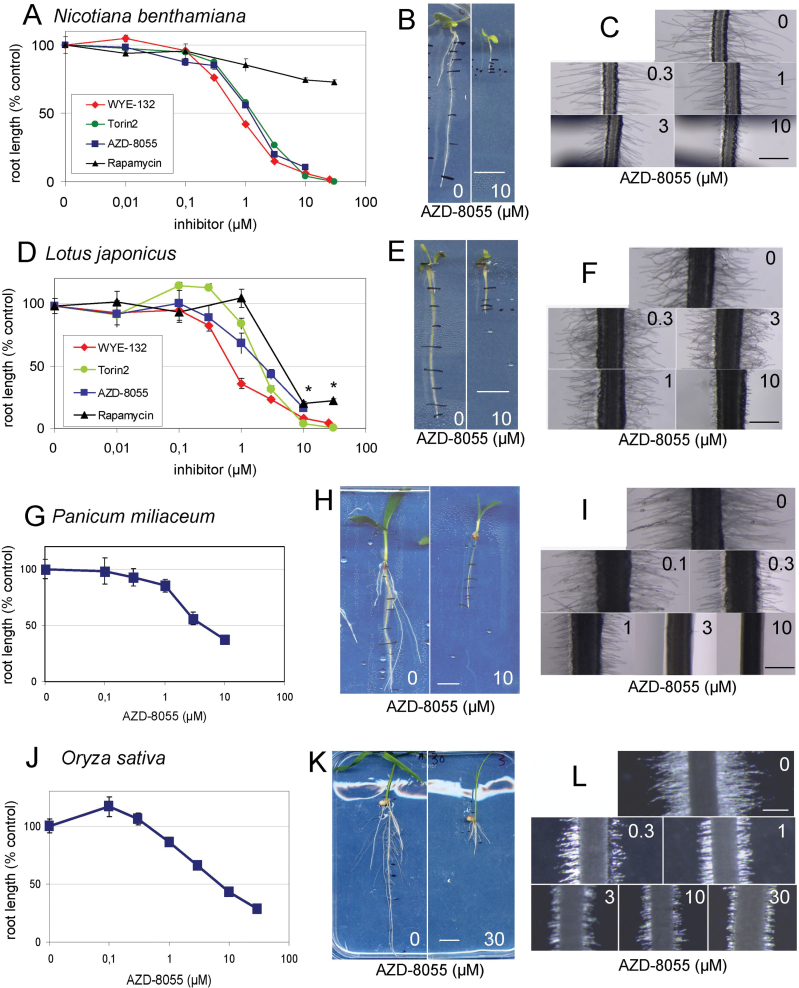
Dose–response curves for AZD-8055, WYE-132, Torin2, and rapamycin on root growth, and root hair and whole-plant phenotypes in divergent angiosperms: *N. benthamiana* (A–C), *L. japonicus* (D–F), *P. miliaceum* (G–I), and *O. sativa* (J–L). (A, D, G, J) Dose-dependent inhibition of root growth by AZD-8055, WYE-132, and Torin2 (A, D) or AZD-8055 (G, J). Asterisks indicate concentration of rapamycin resulting in precipitates observed in the plates and precluding clear conclusions. (B, E, H, K) Pictures of whole plants at selected concentrations of AZD-8055. (C, F, I, L) Pictures of roots in the differentiated area of plants grown on different concentrations of AZD-8055. Bars, 1cm (B, E, H, K), 500 μm (C, F, I, L). (This figure is available in colour at *JXB* online.)

We observed that AZD-8055, WYE-132, and Torin2 inhibited the primary root growth of both *N. benthamiana* and *L. japonicus* in a dose-dependent manner (Fig. 4A, B, D, E). Interestingly, more drug was necessary to inhibit the growth of these plants compared with *A. thaliana*, but, as for *A. thaliana*, WYE-132 inhibited *N. benthamiana* and *L. japonicus* root growth at a lower GI_50_ than AZD-8055 and Torin2 (Figs 1A–C and 4A, D). This suggested that the relative efficiency of these drugs to inhibit TOR is conserved between these species. Indeed, the growth of the aerial part of all species tested, as illustrated with *A. thaliana* (Fig. 1H) and *N. benthamiana* (Supplementary Fig. S6 at *JXB* online), was delayed in a dose-dependent manner. Furthermore, experiments prolonged for up to 2 weeks indicated that asTORis robustly inhibited *A. thaliana* and *N. benthamiana* growth with time and in a dose-dependent manner (Supplementary Fig. S7 at *JXB* online). For *P. milliaceum* and *O. sativa*, which have more complex root architectures, including seminal roots, we recorded the length of the whole root system. Again, root growth inhibition by AZD-8055 was observed in a dose-dependent manner but with a higher GI_50_ than for the Rosids tested (Fig. 4G, H, J, K). A slight stimulation of root growth at low doses occurred for AZD-8055 with *O. sativa* (Fig. 4J) and Torin2 with *L. japonicus* (Fig. 4D). This is typical of hormesis, a universal but still poorly explained phenomenon of growth stimulation at low doses or under slight stress that has been described in plants (Calabrese and Blain, 2009). This indicated that more than one drug should be tested in pharmacological studies in plants. Noticeably, the short root hair phenotype that we observed in *A. thaliana* (Fig. 3F) was also observed in all species (Fig. 4C, F, I and L). Therefore, asTORis inhibited root growth in a dose-dependent manner in the five divergent angiosperms species that we tested in this study, indicating that they could probably be used as an inhibitor of TOR in most angiosperms. Although concentrations inhibiting growth in plants are generally higher than in mammalian cell cultures, a phenomenon probably related to the dynamics of influx/efflux of drugs in plant roots and/or to the root morphology, the conserved short root hair phenotype and the similar progression of the dose–response curves suggest that root growth inhibition by asTORis occurs through the similar mechanism of TOR inhibition in all species tested.

## Discussion

Inhibition of TOR *in planta* has been challenging since the first identification of TOR in *A. thaliana* 10 years ago (Menand *et al.*, 2002; Dobrenel *et al.*, 2011). Indeed, several distinct strategies have been developed involving transgenic plants because of the absence of a clear-cut dose-dependent effect of rapamycin. Here, we showed that several ATP-competitive inhibitors of TOR recently developed for animal cells efficiently inhibited *A. thaliana* growth. All but two (Torin1 and WYE-354) stably inhibited the growth of *A. thaliana*. We did not test high concentrations of Torin1, as it is poorly soluble in DMSO and the homogeneity of our experimental conditions would have been compromised. With regard to WYE-354, we observed a relaxation of inhibition and precipitates occurring during the growing period. This illustrated that the solubility of those compounds together with the stability and bioavailability (Golding *et al.*, 2009; Liu *et al.*, 2011) are key points that have to be analysed carefully in plant pharmacology studies.

Several groups have recently reported that asTORis, which inhibited mTOR but not PI3-K, provide a new pharmacological approach to selective mTOR inhibition (Guertin and Sabatini, 2009; Feldman and Shokat, 2011). Noticeably, we have shown here that the decrease in GI_50_ concentrations for growth inhibition by second-generation asTORis was 10 to 100 times slower compared with first-generation ones (Fig. 1A, C and Table 1). This is agreement with the results reported for mammalian cells. In addition, we showed that three second-generation asTORis, AZD-8055, WYE-132, and Torin2, induced haplo-insufficiency of the *TOR*/*tor-1* heterozygote, i.e. the GI_50_ values for these asTORis were twice as low for the *TOR*/*tor* heterozygotes compared with the WT. AsTORis are highly selective to mammalian TOR compared with tens or hundreds of kinases tested including PI3Ks and other PIKKs, which are the closest structurally related kinases (Table 1 and references therein). Conversely, inhibitors of PI3Ks are barely selective to mTOR as exemplified by LY294002 (Gharbi *et al.*, 2007; Zhang *et al.*, 2011). Indeed, several asTORis including those used in our study have been developed from the lead compound PI-103, a dual inhibitor of PI3K/mTORC1 (Zhang *et al.*, 2011). It is worth mentioning here that asATMis do not inhibit growth at concentrations that are active in plants (Amiard *et al.*, 2010), showing that they are also specific in plants and probably do not compete with ATP in TOR. Due to conservation of the TOR protein, overall our data strongly support the suggestion that AtTOR is a target of asTORis *in planta*. Therefore, the haplo-insufficiency of AtTOR heterozygotes suggests that the amount of TOR protein inside heterozygote cells is twice as low as in the WT. With the stoichiometry observed, we hypothesize that half the amount of AtTOR protein is still sufficient to ensure heterozygote growth that is undistinguishable from the WT without asTORis. Altogether, our data suggest that animal and plant TOR proteins have a similar sensitivity to asTORis at the enzymatic level.

The range of concentrations of asTORis that inhibit growth is generally higher in plants than in mammalians cells (Table 1 and references therein) but much less than in yeast (Liu *et al.*, 2012*b*). The drug content in tissues is the result of the equilibrium between the drug concentration in the medium and in the tissue, and is a function of drug uptake, drug metabolism, drug efflux, and its ability to inhibit all complexes of the targeted protein. In our study, *A. thaliana* growth was inhibited by GI_50_ concentrations of 0.6 μM AZD-8055, 3 μM KU63794, and 10 μM WYE-354 (Fig. 1A–C). In yeast, growth inhibition by the same compounds was obtained with GI_50_ concentrations between 25 and 75 μM. Moreover, this was obtained by using a triple mutant, which lacks two ABC multidrug transporters and the sterol methyltransferase ERG6, showing the importance of efflux and/or metabolism of xenobiotics. Similarly, the ineffectiveness of Torin1 in yeast was probably due to a intracellular concentration that was too low, caused by export by efflux carriers (N.S. Gray, personal communication). Therefore, a number of Torin1 analogues were screened for inhibition at a dose of 10 μM, and QL-IX-55 was selected (Liu *et al.*, 2012*a*). The GI_50_ concentration for QL-IX-55 was nevertheless still higher than 10 μM with the WT and 163nM with the triple mutant, illustrating efflux or metabolism of xenobiotics. It is likely that plants have also efflux and metabolism mechanisms that would detoxify part of asTORis (Kreuz *et al.*, 1996). This would explain the higher concentrations required to inhibit plant growth compared with mammalian cells and the different GI_50_ concentrations of asTORis for inhibition of angiosperm species. In addition, this would explain at least partially why rapamycin barely inhibits plant growth at 10 μM, even in FKBP-overexpressing lines, when inhibition occurs in the nanomolar range in yeast and mammalian cells (Sormani *et al.*, 2007; Ren *et al.*, 2012; Xiong and Sheen, 2012). In addition, the absence of a clear-cut dose-dependence effect of rapamycin on plant growth compared with asTORis probably reminds us that the sole inhibition of TORC1 but not all TOR complexes in mammals is a situation that might also occur in plants, underlining the potency of asTORis in both cases. Lastly, the dose–response curve of *O. sativa*/AZD-8055 and *L. japonicus*/Torin2 had an inverted U shape that is characteristic of hormesis (Calabrese and Blain, 2009). This indicates the importance of careful examination of the effect of several inhibitors on growth.

S6K is a conserved indicator of the TOR kinase activity that is phosphorylated at the T449 of AtS6K. It has been recognized by an antibody developed against the human S6K1 T(P)-389 (Xiong and Sheen, 2012). In another study, 15-d-old plants incubated for a couple of hours with 200nM Torin1 were associated with a decrease in AtS6K1 phosphorylation in extracts of the AT7 line that overexpress the cauliflower mosaic virus translation reinitiation factor TAV (Schepetilnikov *et al.*, 2011). As the AT7 line was supposed to have a constitutive level at least eightfold higher that WT, the endogenous level of the phosphorylated AtS6K was barely detected in the WT. More recently, the phosphorylation of S6K1 T449 was shown to be inhibited when 100nM of Torin1 was added for 30min to transfected protoplasts transiently expressing AtS6K1–FLAG (Xiong *et al.*, 2013). These concentrations are close to those used for mTORC1 and mTORC2 inhibition (Thoreen *et al.*, 2009). If both examples illustrate the inhibition of AtTOR by Torin1, they also show that the endogenous levels of AtS6Ks are barely detectable with antibodies against human S6K in WT plants as already shown (Xiong and Sheen, 2012). The methodology of detection of endogenous plant S6Ks and their phosphorylated counterpart in WT plants has to be improved. For instance, it would be useful to develop antibodies against plant S6Ks and/or more sensitive detection methods such as ELISA (Ren *et al.*, 2012).

The dose-dependent effect of asTORis on *A. thaliana* growth inhibition has allowed us to analyse in detail the progressive process of root growth delay and its consequences on organ development. As the asTORis dose increases, we observed the progressive diminution of the length of (i) the MZ; (ii) the division zone in the MZ; (iii) epidermal cells in the EZ; and (iv) root hair cells. Concomitantly, the density of cells that expressed the marker of the G2/mitotic phase, CYCB1;1–GUS, was unchanged. The effect of asTORis proceeded through a reduction in the number of MZ cells concomitant with their differentiation. The unchanged density of G2-marked cells when MZ size decreased indicated that the asTORis probably do not act on the G2 phase. It would be interesting to examine whether the other cell-cycle phases, and particularly the G1 and S phases, change. Indeed, during revision of this manuscript, a biochemical link between AtTOR inhibition and a marker of the S phase, transcription factor E2Fa, was reported in *A. thaliana* leaf protoplasts (Xiong *et al.*, 2013). As our assay dealt with roots shifting from growth to inhibition of growth in the presence of asTORis and as the above-mentioned assay used protoplasts that are shifting from ‘quiescence’ of leaf cells to potential proliferation of protoplasts, it is difficult to speculate. It would certainly be interesting to follow the phosphorylation status of E2Fa in both situations in order to decipher the link between the cell cycle and TOR.

Along the root, no change in the patterning of cell lines such as hair-bearing and non- hair-bearing cells or of the patterning of root hair emergence was observed. This suggests that root growth inhibition by asTORis is a ‘mild’ process that does not trigger tissue dislocation, probably through a ‘mild’ effect on the cell cycle among other putative functions. Indeed, the size of the plants at the time of transfer did not change the dose–response curve. The cyclin-dependent kinase inhibitor roscovitine and the protein synthesis inhibitor cycloheximide did not phenocopy the asTORi-induced phenotype. This indicated that asTORis phenotypes would not be exclusively and/or directly due to inhibition of the cell cycle or translation. This suggests that the plant TOR inhibition phenotype results in coordinated and multifaceted changes.

Altogether, these phenotypes illustrate a slowdown in growth dynamics caused by TOR inhibition, as evidenced by a reduction in the number of divisions undergone by meristematic cells and reduced elongation as they approach the transition zone. Furthermore, the elongation of these prematurely differentiated cells was also decreased and subsequently the root hairs were also shortened. The development of the aerial part of all species studied was also slowed by asTORis, and the number and size of leaves decreased concomitantly with root shortening. However, no stress phenotypes could be observed. Therefore, the overall process of TOR inhibition is likely to operate by triggering a signal of growth delay or arrest that homogeneously affects all cells and does not interfere with the cell fate. The strength of this reversible slowdown signal is a function of the concentration of the asTORi and is of primary importance to further studies.

As AtTOR is expressed in the MZ (Menand *et al.*, 2002), the use of asTORis opens new possibility of studying the connection between hormone signalling, cell fate, and the AtTOR pathway. Indeed, the asTORis-induced reduction of MZ resembles the effect of an increase in cytokinins and a decrease in gibberellic acid (Dello Ioio *et al.*, 2007; Ubeda-Tomas *et al.*, 2009). Assuming that the accumulation of hormones transporters and hormone synthesis are not disturbed, whereas proliferation slows down, the premature differentiation of asTORi-treated cells may be caused by an increase and shortening of the hormone gradient along the root (Muraro *et al.*, 2013). This hypothesis is supported by the recent report that auxin and cytokinin signalling does not require AtTOR signalling and mitochondrial energy relay (Xiong *et al.*, 2013). Lastly, as the PI3K inhibitor LY294002 generates a root phenotype close to asTORis, we suggest that, as in mammals (Laplante and Sabatini, 2012), PI3K may act upstream of AtTOR to integrate hormone signalling. However, more in-depth studies are required, as LY294002 was found not to be exclusively selective for the PI3Ks (Gharbi *et al.*, 2007).

A reduction in the MZ and root hair length was also reported in FKBP12-overexpressing lines grown on 10 μM rapamycin (Ren *et al.*, 2012), sharing part of the phenotypes with asTORis, although a rapamycin dose dependency was not reported. This argues in favour of the efficiency of asTORis in plants with the advantage of a clear-cut dose–response dependency of growth inhibition and tissue morphology in a swift and reproducible way. Therefore, asTORis allow inhibition of plant TOR without the production of chimaeric transgenic lines and/or RNA-silenced lines. Therefore, asTORis should help identify new targets of plant TOR kinase and establish the structure of the plant TOR pathway and its specific interconnection with cell energy status and hormone signalling. The finding that asTORis efficiently inhibit growth of different plant species shows that they could be used to decipher the degree of conservation of the TOR pathway among angiosperms.

## Supplementary Material

Supplementary Data
