# Supplementary material for: ATP-competitive mTOR kinase inhibitors delay plant growth by triggering early differentiation of meristematic cells but no developmental patterning change
Source: J Exp Bot. 2013 Aug 20;64(14):4361–74. doi: 10.1093/jxb/ert242 (PMC3808319; doi:10.1093/jxb/ert242)
Supplement: Supplementary Data [file supp_64_14_4361__index.html]

ATP-competitive mTOR kinase inhibitors delay plant growth by triggering early differentiation of meristematic cells but no developmental patterning change — ATP-competitive mTOR kinase inhibitors delay plant growth by triggering early differentiation of meristematic cells but no developmental patterning change — Supplementary Data 

# ATP-competitive mTOR kinase inhibitors delay plant growth by triggering early differentiation of meristematic cells but no developmental patterning change

## 

Data files

**Files in this Data Supplement:**

- Supplementary Data - Supplementary Data
