# Supplementary material for: ATP-competitive mTOR kinase inhibitors delay plant growth by triggering early differentiation of meristematic cells but no developmental patterning change
Source: J Exp Bot. 2013 Aug 20;64(14):4361–74. doi: 10.1093/jxb/ert242 (PMC3808319; doi:10.1093/jxb/ert242)

Supplementary Figure 1. Sequence alignment of the kinase domain of mTOR (*Mus musculus* TOR, accession NP_064393.2), AtTOR (*A. thaliana*, accession Q9FR53), and ScTOR1 (*S. cerevisiae*, accession CAA52849.1). The alignment was done with Multalin (http://multalin.toulouse.inra.fr/multalin/). Red letters indicate 90% consensus value and blue letters indicate 50% consensus value. Green colors bars above each row highlight the aminoacid residues involved in the formation of the ATP binding pocket based on molecule modeling, according to Liu *et al.*, 2012. These aminoacid residues are highly conserved between mTOR and AtTOR. The kinase domain is also highly conserved with a percentage of identity of 71,9% between AtTOR and mTOR, and 65,9% between ScTOR1 and mTOR.

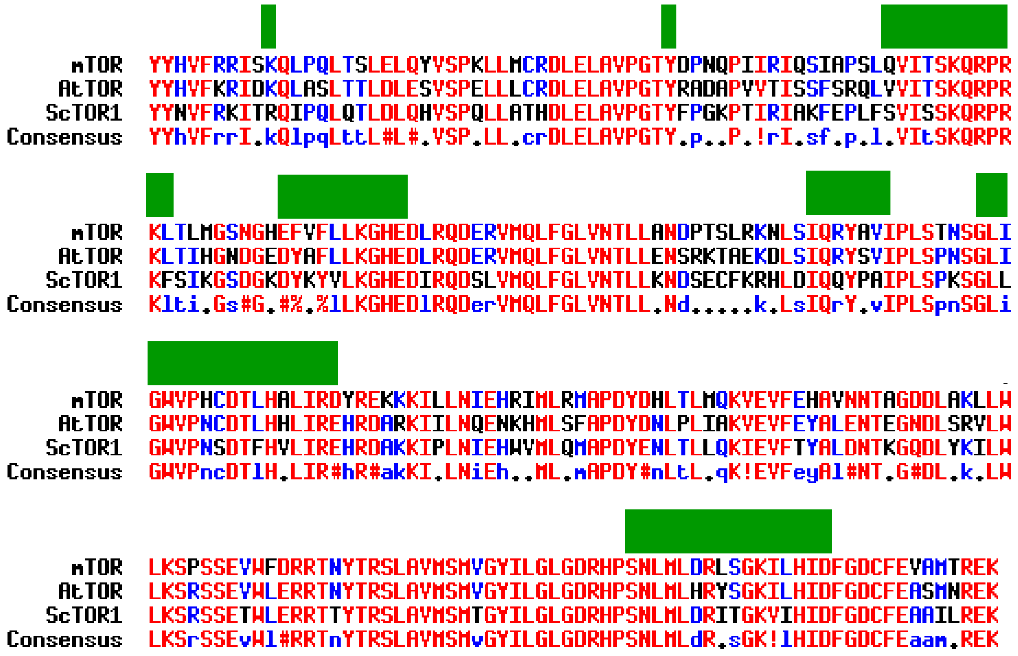

Supplementary Figure 2. Effect of agar and DMSO on the kinetics of primary root growth after transfer.

For this experiment and for all other *A. thaliana* (Col 0) root growth experiments, seeds were sown on agar plates and incubated at 4°C for 2 days. 3 day-old vertically germinated *A. thaliana* plants without drug were manually transferred to plates containing media solidified with 0.8% agar (Sigma Aldrich, ref. A9799 (A)) or 0.4% phytagel (ref. Sigma Aldrich, P8169, (B)), sealed with one layer of micropore tape (ref. 1530, 3M, VWR, France) and vertically grown for 6 days on medium containing or not DMSO at a final concentration up to 0.1%. For daily root length measurements, plates were labelled everyday at the tip of the root. The growth of the roots was swift and vigorous with solidified phytagel compared to agar (C). We used therefore phytagel containing media for all species.

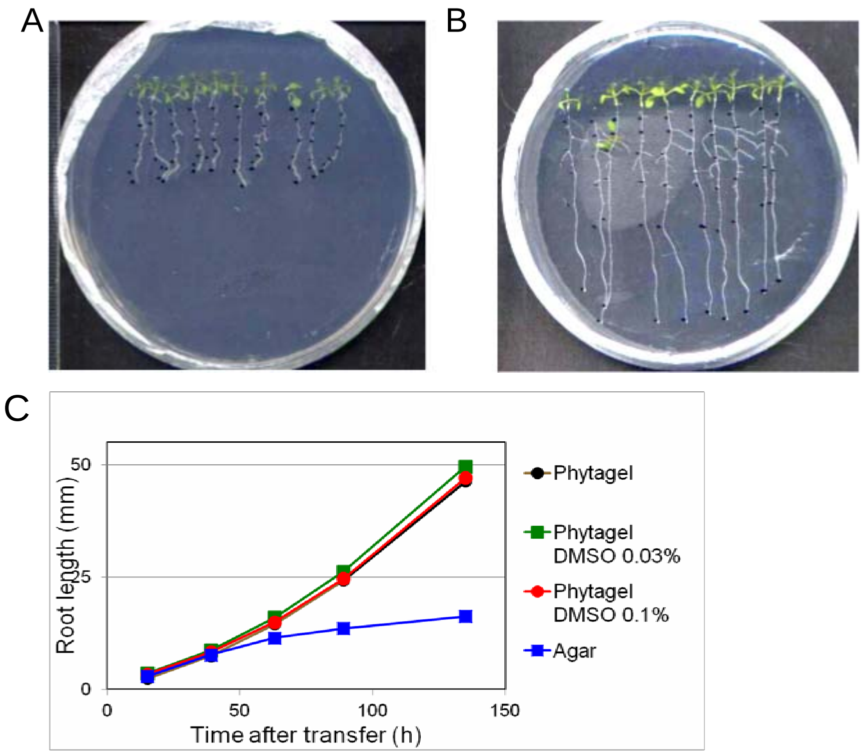

Supplementary Figure 3. Dose dependent effect of cycloheximide and roscovitine on *A. thaliana* col-0 roots. Assay and measurements were as in Figure 1.

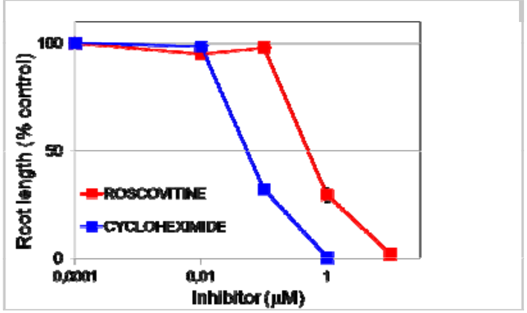

Supplementary Figure 4. Time course of *A. thaliana* root growth inhibition by inhibitors of different cell targets. AZD-8055 (AZ) and QL-IX-55 (QL) were asTORis potent for mammalian TOR and yeast TOR2. LY294002 (LY) is an inhibitor of PI3Ks. Cycloheximide (CX) inhibits protein synthesis and Roscovitine (RO) inhibits CDKs. Experiments were done as in Fig. S3.

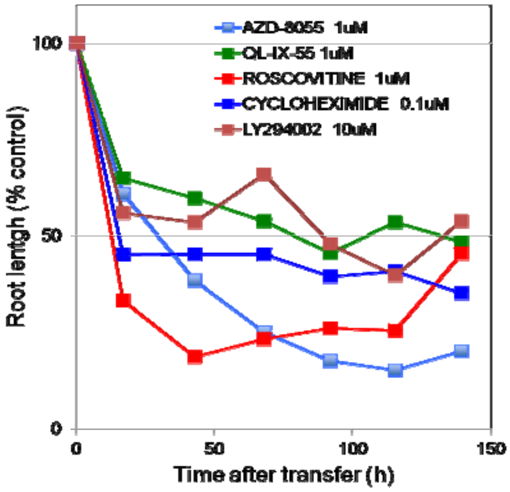

Supplementary Figure 5. Pictures of *A. thaliana* root epidermal cells in the differentiated zone of roots of plants grown on AZD8055 compared to DMSO. The arrows indicate the position of emergence of root hairs, which is not affected in AZD-8055 treated roots. The experiment was done as in Figure 3. Scale bar, 20 µm.

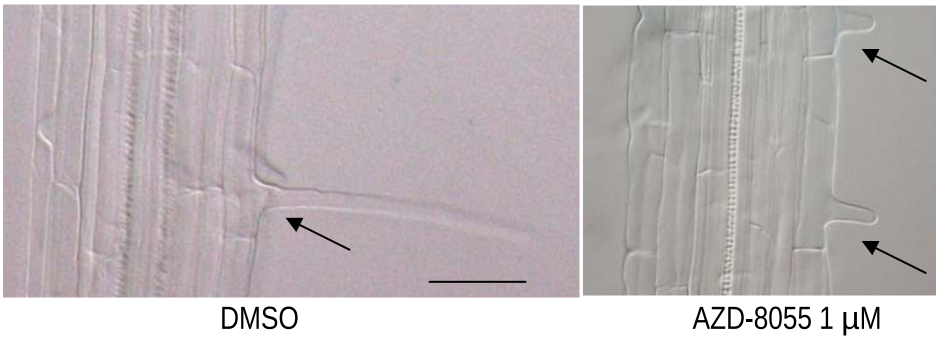

Supplementary Figure 6. Dose-dependent inhibition of the growth of the aerial part of *Nicotiana benthamiana* by asTORis. Pictures of the aerial part of plants 6 days after transfer on media containing different concentrations of AZD-8055 (A), WYB-132 (B) or Torin2 (C). Scale bar, 2 mm.

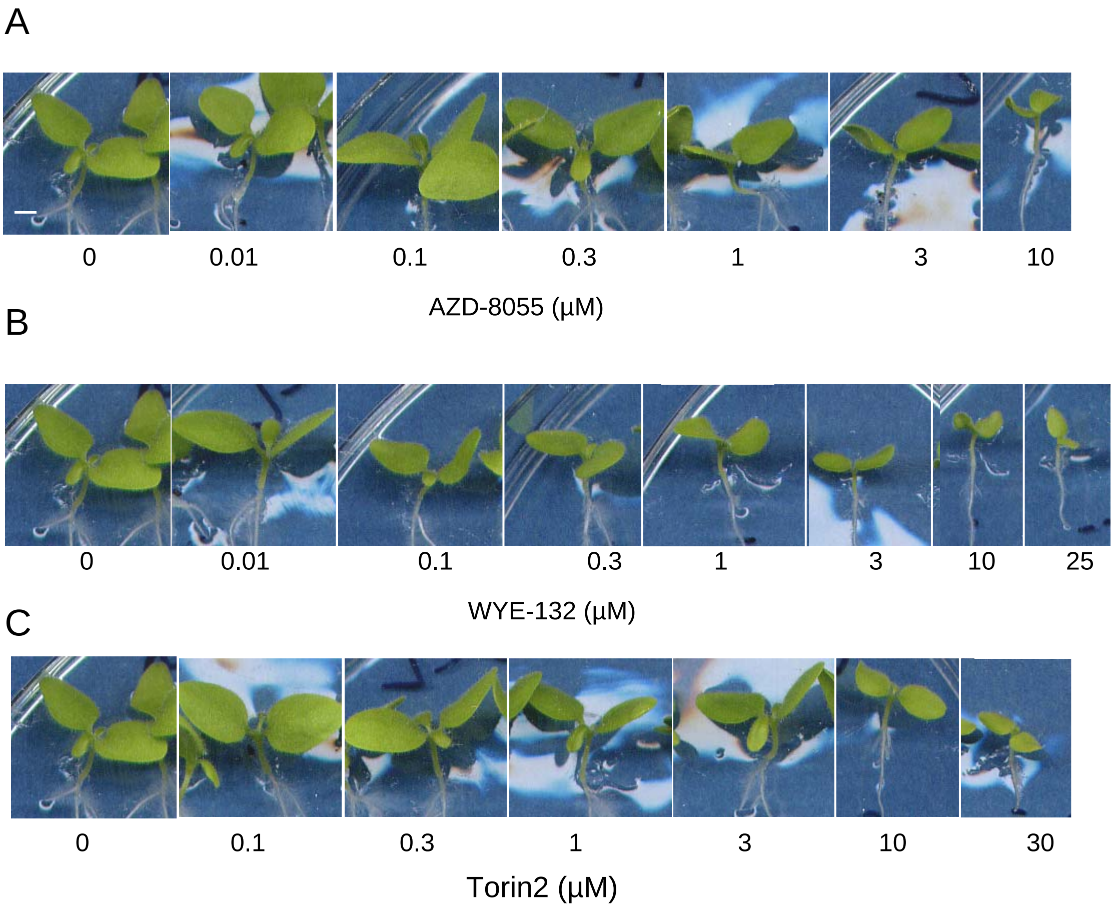

Supplementary Figure 7. Time course of primary root growth inhibition by asTORis on a 2.5 weeks period. (A) Inhibition of *A. thaliana* root growth by different concentration of AZD-8055. (B) Inhibition of *Nicotiana benthamiana* root growth by WYE-132 and AZD-8055.

A

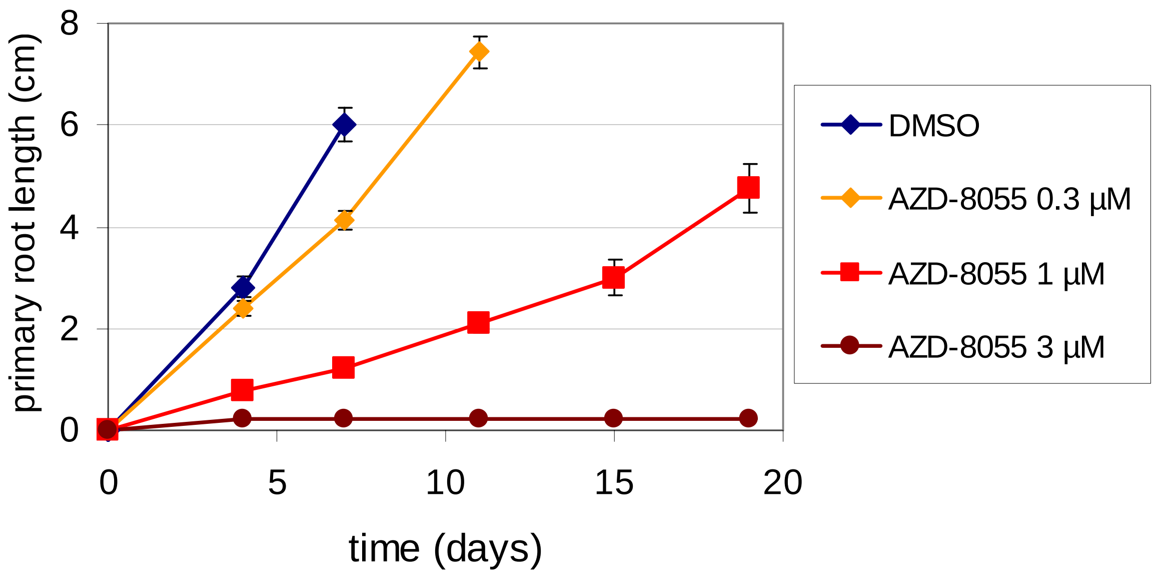

B

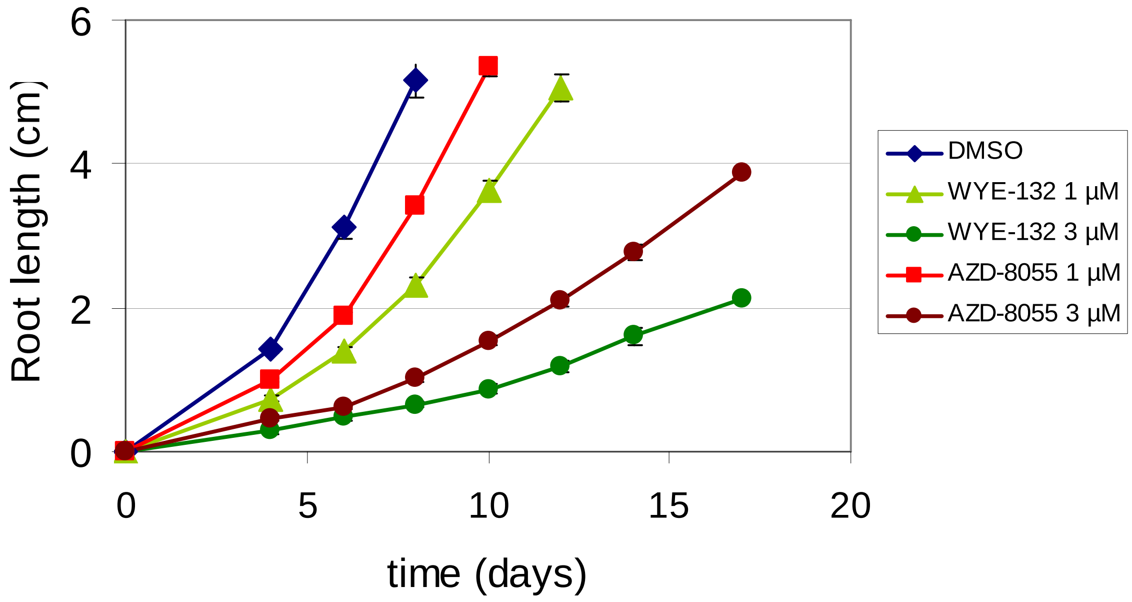

Supplement: Supplementary Data [file supp_ert242_jexbot098137_file001.pdf]
